# Characterization of activated bentonite clay mineral and the mechanisms underlying its sorption for ciprofloxacin from aqueous solution

**DOI:** 10.1007/s11356-020-09267-1

**Published:** 2020-06-10

**Authors:** Ali Maged, Sherif Kharbish, Ismael Sayed Ismael, Amit Bhatnagar

**Affiliations:** 1 Department of Environmental and Biological Sciences, University of Eastern Finland, P.O. Box 1627, FI-70211 Kuopio, Finland; 2 Geology Department, Faculty of Science, Suez University, El Salam City, Suez Governorate 43518 Egypt

**Keywords:** Natural bentonite, Acid activated bentonite, Ciprofloxacin, Sorption, Mechanism

## Abstract

The presence of emerging pollutants such as hazardous chemicals, pharmaceuticals, pesticides, and endocrine-disrupting chemicals in water sources is a serious concern to the environment and human health. Thus, this study focused on exploring the interaction mechanisms between ciprofloxacin (CIP) (antibiotic) and clay (a low-cost adsorbent) during sorption process. Acid activation technique was opted for modifying natural bentonite (NB) to enhance the adsorptive removal of CIP from water. The BET surface area analysis revealed that acid-activated bentonite (AAB) possessed more than two fold higher surface area as compared to NB. Combining pH_zpc_ measurements, effect of solution pH and CIP speciation revealed that CIP sorption onto bentonite is highly dependent on solution pH. Kinetic studies confirmed that CIP sorption mechanism was chemisorption which included ion-exchange and surface complexation mechanisms. The mechanism of CIP sorption onto AAB was successfully explored with the assistance of characterization techniques. Maximal monolayer sorption capacity of AAB was found to be 305.20 mg/g, compared to 126.56 mg/g for NB. Reusability studies demonstrated that AAB could be reused successfully up to 5 cycles. Furthermore, column studies showed satisfactory results confirming that AAB can be successfully used in continuous mode for practical applications.

**Electronic supplementary material:**

The online version of this article (10.1007/s11356-020-09267-1) contains supplementary material, which is available to authorized users.

## Introduction

The removal of emerging contaminants from aquatic ecosystems has gained much interest due to their associated environmental risks and impact on human and animal health. Emerging contaminants (ECs) are defined as any chemicals or pollutants which are presented in a very low concentration and not continuously monitored in the aquatic environment (Rodriguez-Narvaez et al. 2017). Despite their low concentration, ECs cause adverse ecological effects on human and animal health (Zhou et al. 2020). The main groups of ECs are pharmaceutical compounds, pesticides, dyes, surfactants, detergents, and personal care products (Li et al. 2019). Antibiotics are one of the pharmaceutical compounds that are widely consumed either for treatment or prevention of diseases and/or infections, such as norfloxacin, sulfamethoxazole, ciprofloxacin, macrolides, and tetracyclines (Carneiro et al. 2019).

Ciprofloxacin (CIP) belongs to the fluoroquinolones (FQS) antibiotic group (Li et al. 2016), marketed and used worldwide for several bacterial infections treatment in humans and animals (Carabineiro et al. 2011). Due to the incomplete metabolization of CIP in human and animal bodies, CIP continuously emerges in the aquatic environment. CIP was detected in the range of nanograms per liter to micrograms per liter in both ground and surface water (Martins et al. 2008; Genç et al. 2013). However, CIP was detected at much higher concentrations in the effluents of the drug production facilities (up to 50 mg/L) and hospital wastewaters (up to 150 μg/L) (Larsson et al. 2007; Li et al. 2016). The presence of low CIP concentrations in the aquatic environment can lead to the growth of antibiotic resistance. Thereafter, these concentrations cause the normally effective antibiotics to fail completely in curing illnesses (Bhandari et al. 2008; Diwan et al. 2010; Homem and Santos 2011). Furthermore, the irrigation of plants with antibiotic-contaminated water leads to the uptake of antibiotics by plants and then antibiotics exposure to the food chain (Bagheri et al. 2020).

Nevertheless, only a few studies have dealt with the elimination of CIP from water, compared with other antibiotics. Several methods have been used for CIP removal which include chemical oxidation and electrochemical (Xiao et al. 2010), oxidation by chlorination (Li and Zhang 2012), ozonation (Nasuhoglu et al. 2012), photolytic and photocatalytic treatment (Vasquez et al. 2013), photo-Fenton oxidation processes (Sun et al. 2009), and enzymatic degradation and biological treatment (Dorival-García et al. 2013), and adsorption (Chen et al. 2015; Wang et al. 2016). Most of these techniques are known for high execution expenses or less ability to accomplish respectable water quality, except for adsorption. Adsorption process has shown good performance in water decontamination against different pollutants, e.g., antibiotics (Maged et al. 2020) and heavy metals (Abu-Danso et al. 2020). One of the advantages of adsorption is the ability to scale up the process, without producing any by-product in the environment during treatment. Moreover, many adsorbents in either batch or continuous flow process (after saturation) possess the ability to be recovered and reused for many cycles. In this regard, many studies have been conducted using natural, commercial, and composite of different materials for CIP removal from water such as kaolinite (Li et al. 2011), halloysite (Duan et al. 2018), schorl (Yin et al. 2018), activated carbon (de Oliveira Carvalho et al. 2019), graphene oxide/calcium alginate (Wu et al. 2019), synthesized nanoceria (Rahdar et al. 2019), and nano-sized magnetite (Rakshit et al. 2013).

Clay minerals such as bentonite, kaolinite, and illite are naturally abundant adsorbent materials on the earth, while having significant historical impacts on human civilization development. Clay minerals play an important role for environmental protection, when utilized for the hazardous substances transfer and storage, owning to their cation exchange capacity (CEC) (Felycia et al. 2015). Clay minerals are considered as the most effective adsorbents due to their considerable specific surface area, pore-volume, negative surface charge, and hydrophilic surface (Srinivasan 2011; Uddin 2017).

Bentonite is an inorganic 2:1 type clay mineral, which is mainly constituted of montmorillonite (Maged et al. 2020). Due to the excellent sorption, physical and chemical properties of bentonite (i.e., CEC, porosity, particle size, and surface area), bentonite is considered as the best candidate for sorption of different kinds of emerging pollutants (Genç and Dogan 2015). Furthermore, the ability of modifying bentonite by different kinds of modifiers such as organic or inorganic chemicals and acid or alkaline solutions can result in the enhancement of sorption capacity for different kind of pollutants.

In the present study, the potential of raw (NB) and acid-activated bentonite (AAB) clay for ciprofloxacin (CIP) removal from aqueous solution was investigated. Although the removal of CIP by bentonite and activated bentonite clay has been studied to a certain extent, previous studies lack (i) a detailed characterization of the adsorbents (raw and modified forms), (ii) study of the removal of pollutants using clay in a continuous flow system (column) in addition to the batch studies, and (iii) successful exploration of the possible sorption mechanisms, based on the experimental data and the characterization results after sorption. Therefore, in the present study, we have provided the detailed characterization of the adsorbent to get an insight of the physico-chemical properties of the adsorbent, which play an important role in the CIP sorption. Moreover, we also conducted column studies, besides batch studies, to know the practical applicability of the prepared adsorbent. Finally, we discussed the possible sorption mechanisms, based on the experimental data and the characterization results after sorption. Adsorption studies were conducted under ambient and controlled pH conditions. Different experiments were conducted to get insight into the interaction between CIP molecules and bentonite clay. Furthermore, well-known kinetic models were applied to predict the mechanism of CIP sorption onto the raw and modified bentonite. In order to understand the sorption process, three different isotherm models were studied.

## Materials and methods

### Clay mineral

The natural 2:1 clay mineral (two silica tetrahedral sheets and one alumina octahedral sheet) sample, selected for this study, is a natural bentonite clay. The natural bentonite clay samples were collected from Matrouh city, Egypt. The lump raw bentonite (500 g) was dried (70 ± 1 °C) in an air oven, crushed, ground, and sieved to fine powder (> 160 μm). The CEC of the sample was determined as 81 meq/100 g of clay.

### Chemicals

**Fig. 1:**
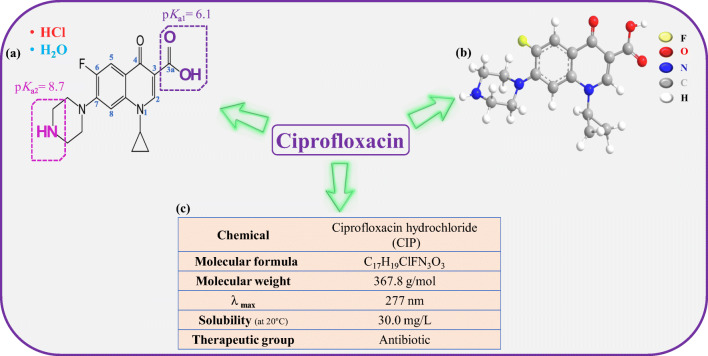
(a) Chemical structure of CIP depicting ionizable groups and their dissociation constant (pK_a_) values, (b) 3D structure of TC, and (c) CIP properties

CIP hydrochloride (purity > 98%) was purchased from Sigma-Aldrich. The important CIP properties are given in Fig. 1. Hydrochloric acid (HCl) was used as an acid activating agent for the natural bentonite. Sodium hydroxide (NaOH), hydrogen peroxide 30% w/w (H_2_O_2_), sodium chloride (NaCl), sodium acetate (Na-Ac), and acetic acid (Ac-Ac) were of analytical grade (Sigma-Aldrich) and used without further treatment.

### Adsorbent preparation

#### Pre-treatment of bentonite

The natural bentonite was purified in the laboratory to remove calcite, carbonates, and organic matters by the following protocol: the sample was first repeatedly washed with Milli-Q water and dried after each time. The dried bentonite clay (25 g) was soaked in 400 mL solution containing Na-Ac (0.1 N) and Ac-Ac adjusted to pH ≈ 5.0. The mixture was stirred for 12 h at 70 ± 1 °C with magnetic stirring followed by overnight stirring at room temperature. During stirring, 100 mL of H_2_O_2_ solution was gradually added to the mixture. The suspension was then centrifuged and washed thrice with 0.01 N NaCl solution followed by three times with Milli-Q water. Thereafter, the residue was dried at 105 ± 1 °C for 24 h using a laboratory air oven. The purified sample (labeled as “NB”) was ground, sieved (100 μm), stored in glass vial, and placed in a desiccator until further use.

#### Acid activation of bentonite

In order to optimize the acid activation of NB, the effect of different acid concentrations was investigated (the optimization of acid activation is given in the supplementary material, section S1). HCl solution was used as bentonite modifier. The activation procedure was done according to the following steps: (a) NB was added to HCl solution (0.5 M). NB weight to volume ratio was 1:2, (b) the mixture was placed in a water bath at 70 ± 1 °C for 4 h, (c) the precipitation was rinsed several times with milli-Q water and then AgNO_3_ test was carried out to eliminate free Cl^-^ anions, (d) the slurry was dried in an oven at 60 °C for 24 h. (e) Finally, the acid activated bentonite (labeled as AAB) was ground and sieved using a 100-μm sieve and stored until further use.

### Physicochemical analysis

The NB and AAB samples were characterized by various characterization techniques. The details of methodology and instrumentation used are given in the supplementary material (section S2).

### Batch sorption studies

The batch sorption experiments were performed with NB and AAB in order to determine their potential towards CIP removal from water. Optimization studies were carried out by dispersing 0.2 g/L of the adsorbent using a known volume of CIP solution (30 mg/L) in a dark 100-mL capped glass bottle. The sorbent-sorbate solutions (in duplicate) were shaken (200 rpm) in an orbital shaker at ambient temperature. To get insights into the effect of solution pH on CIP sorption, the experiments were performed in the pH range of 3.0–9.0. To assess the optimum adsorbent dosage for sorption experiments, the effect of various adsorbent dosages (0.1–0.8 g/L) was studied. The sorption kinetic studies were carried out with different interaction times (0–240 min). To study the sorption isotherms, the effect of initial CIP concentrations (5–200 mg/L) was investigated. The effect of ionic strength (0.05–0.5 M) on the sorption process was also investigated. Before NB and AAB were added, the solution pH was adjusted using NaOH (0.1 M) and/or HCl (0.1 M). After equilibration time, the sorbent-sorbate solution was separated using syringe driven filter (0.45 μm). All the experiments were done in duplicate. The amount of CIP sorbed onto NB and AAB (*q*_*e*_; mg/g) and percentage removal (*R*; %) were calculated using the following equations (Eqs. (1–2)):

1 $$ {q}_e=\frac{C_i-{C}_e}{m}\ast V $$

2 $$ R\ \left(\%\right)=\frac{C_i-{C}_e}{C_i}\ast 100 $$

#### Batch regeneration studies

Reusability study is helpful in illustrating the regenerating capability of the bentonite clay. The regeneration experiments in batch mode were performed with NB and AAB based on the aforementioned batch optimization studies. Two eluents (0.1 M NaOH and 0.1 M HCl) were selected to test the regeneration potential of bentonite. After equilibrium was achieved, the separated adsorbents were washed several times and dried. Thereafter, a known volume of the eluent was agitated with the adsorbent for 4 h. Then, the adsorbent was washed with milli-Q water, dried, and used for the next cycle again. The regenerating experiment was repeated for five consecutive cycles.

### Kinetic and equilibrium isotherm modeling study of CIP adsorption

#### Kinetic modeling

To understand and evaluate the rate and mechanism of CIP removal, the sorption kinetics were investigated. The kinetic experimental data were validated with three well-known kinetic models using non-linear form. The models, namely pseudo-first order (PFO) (Lagergren 1898), pseudo-second order (PSO) (Ho and McKay 1999), and intra-particle diffusion (IPD) (Weber and Morris 1963), were studied for CIP adsorption onto NB and AAB. The detailed information about the models, equations, and abbreviations are given in the supplementary material (section S3.1).

#### Equilibrium isotherm modeling

To get insight into sorbent and sorbate interaction with various initial pollutant concentrations, the sorption isotherms were investigated. The experimental adsorption data of NB and AAB were applied to three isotherm models using non-linear form. These models include two-parameter (Langmuir (Langmuir 1918) and Freundlich (Freundlich 1924)) and three-parameter (Sips 1948) isotherm models. The detailed information about the models, equations, correlation coefficient (*R*^2^), root mean square error (RMSE), and abbreviations are given in the supplementary material (section S3.2).

### Fixed-bed column sorption studies

#### Column preparation

A laboratory-scale glass column with an internal diameter of 5 mm and a length of 130 mm was used to conduct fixed-bed column studies with AAB. The column was packed with AAB wedged between a glass wool layer to prevent any loss of adsorbent mass. The column was closed tightly to promote liquid phase distribution. To study the effect of varying column beds, two different loadings of adsorbent (25 and 50 mg) were used. To simulate the effect of influent flow, two different flow rates (1.5 and 3 mL/min) were studied. Likewise, two different CIP concentrations (10 and 20 mg/L) were used to study the effect of influent concentration (C_o_). While running the experiments, CIP effluent samples were collected at different time intervals. Column operation was suspended when no further CIP adsorption was detected, i.e., when the CIP influent and effluent concentrations became similar.

#### Column data analysis

CIP concentration in effluent samples was measured using UV-VIS spectrophotometry. The column performance is characterized by the breakthrough curve. Based on the breakthrough time and shape, the operation and dynamic response of the fixed-bed column sorption can be determined. The breakthrough point (*t*_*b*_, min) was determined as the first observed point when *C*_*t*_/*C*_*o*_ exceeds 0.05. The column exhaustion point (*t*_*e*_, min) was determined when *C*_*t*_/*C*_*o*_ displays a constant value. *C*_*t*_/*C*_*o*_ values were plotted against the time (min) in order to represent the breakthrough curve. From the area under the breakthrough curve, the total CIP adsorbed quantity (*q*_total_, mg) can be calculated using Eq. (3), while Eq. (4) calculates the total effluent volume (*V*_eff_, mL) which passed through the column system. The column’s experimental maximum uptake capacity (*q*_bed_, mg/g) was calculated from Eq. (5). The total CIP molecules delivered to the continuous flow system (*m*_total_, mg) was calculated from Eq. (6). The CIP removal percentage (*RE*, %) was calculated from Eq. (7). The unadsorbed CIP concentration (*C*_eq_; mg/L) by the column system was calculated from Eq. (8) (Chen et al. 2012).

3 $$ {q}_{\mathrm{total}}=\frac{Q}{1000}{\int}_{t=0}^{t=\mathrm{total}}{C}_{ad} dt $$

4 $$ {V}_{\mathrm{eff}}=Q\times {t}_{\mathrm{total}} $$

5 $$ {q}_{\mathrm{bed}}=\frac{q_{\mathrm{total}}}{M_{\mathrm{AAB}}}\kern0.5em $$

6 $$ {m}_{\mathrm{total}}=\frac{C_o\times Q\times {t}_{\mathrm{total}}}{1000}\kern0.5em $$

7 $$ RE\left(\%\right)=\frac{q_{\mathrm{total}}}{m_{\mathrm{total}}}\times 100\kern0.5em $$

8 $$ {C}_{\mathrm{eq}}=\frac{m_{\mathrm{total}}-{q}_{\mathrm{total}}}{V_{\mathrm{eff}}}\times 1000 $$

#### Column regeneration and recycling

After selecting the optimum condition from the column tests, the AAB adsorbent was regenerated by the following procedure: a solution of 0.1 M NaOH was injected into AAB packed column with flow rate of 1 mL/min. The injected solution was kept running until CIP concentration became lower than the detection limit. Next, Milli-Q water was injected into the column in order to eliminate Cl^-^ anions (AgNO_3_ test) from the adsorbent inside the column. Thereafter, the experiment was repeated three times under the same optimization conditions, while the column parameters for each cycle were determined.

### Adsorption mechanism study

In order to get insight into the sorption mechanism between CIP molecules and AAB adsorbent, the characterization (X-ray diffraction (XRD), Fourier transform infrared (FTIR), and scanning electron microscopy (SEM)) studies for AAB before and after CIP sorption were conducted. Based on the batch optimization studies, the saturated AAB samples with CIP were prepared. Thereafter, the saturated AAB samples were dried for 24 h in a laboratory oven at 50 ± 1 °C. Finally, a comparison study between the saturated and unsaturated AAB samples was performed to monitor the changes in the structure, functional groups, and surface morphology.

## Results and discussion

### Characterization studies

#### X-ray fluorescence analysis

The X-ray fluorescence (XRF) analysis was performed in order to determine the chemical compositions of NB and AAB. Table 1 shows that SiO_2_ and Al_2_O_3_ were the main constituents of examined clay samples. The XRF results revealed that the Al_2_O_3_/SiO_2_ ratio was 0.36 and 0.30 for NB and AAB, respectively. These ratios indicated that NB and AAB clay contained the montmorillonite mineral (Abdou et al. 2013). Furthermore, the bentonite samples also contained large quantities of Fe_2_O_3_; however, K_2_O, CaO, MgO, TiO_2_, and P_2_O_5_ were present as minor metallic oxides (Table 1). After the acid activation, the analysis data showed a significant change in the chemical composition in modified bentonite clay, compared to the raw bentonite clay. The acid activation of bentonite caused an increase in the amount of Si^+4^ and Ti^+4^; this increase could be attributed to the remobilization of cations in the octahedral or tetrahedral sites (Venaruzzo et al. 2002). However, the alumina content decreased from 20.04% (NB) to 18.76% (AAB); this decrease could be attributed to the dealumination of sample after the acid attack. Likewise, the decreases in other metal oxides (Fe_2_O_3_ and MgO) contents could be a result of the ion migration from octahedral sheet to exchange position in the structure and subsequently their removal after the acid activation. The acid modification could improve the sorption properties owing to the alteration of the bentonite surface (Djomgoue and Njopwouo 2013).

**Table 1:** The chemical composition of natural (NB) and acid activated bentonite (AAB) sample (% by weight)

Metal oxide (wt%)	Bentonite clay samples	
	NB	AAB
SiO_2_	55.41	60.02
Al_2_O_3_	20.04	18.76
TiO_2_	0.56	0.61
Fe_2_O_3_	8.45	6.27
MgO	2.28	1.41
CaO	0.66	0.10
K_2_O	1.13	1.05
Na_2_O	1.69	1.07
P_2_O_5_	0.11	0.09
L.O.I.	9.63	10.51
Al_2_O_3_/SiO_2_	0.36	0.30

*L.O.I.* loss on ignition

#### X-ray diffraction analysis

**Fig. 2:**
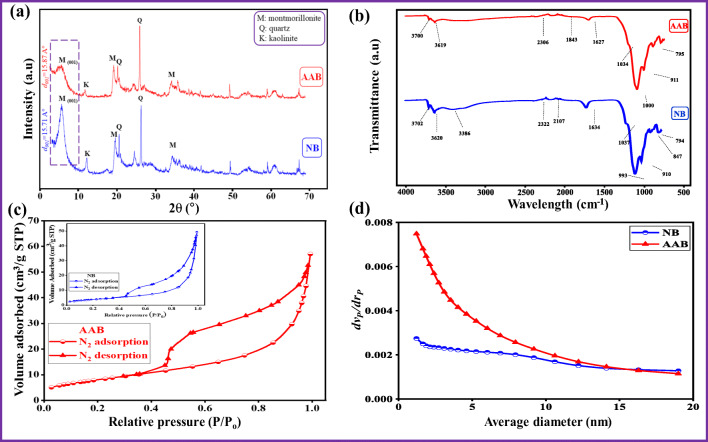
Characterization results of the natural bentonite before and after treatment (a) XRD pattern, (b) FTIR spectra, (c) N_2_ adsorption–desorption isotherm, and (d) BJH pore diameter distribution patterns

The structural and layer spacing changes that occurred in the natural bentonite before and after the acid activation were studied using the X-ray diffraction technique. Figure 2(a) represents the XRD patterns of NB and AAB samples. The typical characteristic peak of NB was detected at a 2*θ* value of 5.62° (*d*_*001*_ spacing of 15.7127 Å). Moreover, NB exhibited a well-defined and very intense “001” peak. From the XRD pattern, both NB and AAB samples contain montmorillonite, quartz, and kaolinite, which confirmed the presence of the smectite phase (Gates 2006; Holmboe et al. 2012). Activation of NB sample with HCl produces significant mineralogical changes which could be seen from the XRD pattern of AAB (Fig. 2(a)). After acid activation, the “001” peak of montmorillonite appeared broad which could be attributed to the partial destruction of the layered structure of the smectite (Falaras et al. 1999). Intensity decreases and width increases of “001” peak in the AAB indicated that the crystallinity of AAB was significantly affected by the acid activation. The AAB sample showed a 2*θ* reflection of “001” peak at 5.60° (*d* spacing of 15.87 Å), which was slightly shifted towards the lower angle with a decrease in the peak intensity compared to parent bentonite clay sample. During HCl treatment of NB, the leaching of metallic constituents from NB did not damage the inherent layered structure of NB. It is clearly observed that there is no other significant difference between the XRD profiles (Fig. 2(a)). Furthermore, it is important to consider that the balance between the acid activation and the structural preservation hold the key for the establishment of optimal adsorbent synthesis.

#### Fourier transform infrared analysis

The FTIR spectra of NB and AAB samples are shown in Fig. 2(b). For natural and activated bentonite, the water molecules (interlayers) and the structural hydroxyl groups in the bentonite clay layers were observed in the region between 3750 and 3500 cm^−1^, whereas the main characteristic silicate bands appeared between 1200 and 700 cm^−1^. Similar findings for bentonite were reported by other authors (Petit 2006; Noyan et al. 2007). Before acid activation (NB), the bands at 3620 and 3386 cm^−1^ are assigned to the O–H stretching vibration of the silanol (Si–OH) groups from the clay (coordinated to octahedral Al^3+^ cations) and HO–H vibration of the adsorbed water molecules on the bentonite surface, respectively (Emmerich et al. 1999), while the band at 1634 cm^−1^ reflects the angular deformation H–O–H bond of interlayer water molecules in the silicate matrix. The sharp band near 1000 cm^−1^ is assigned to stretching vibration of Si–O groups of the tetrahedral layer (Petit 2006). The bands at 910 and 847 cm^−1^ are attributed to Al–Al–OH and Al–Mg–OH, respectively. The band at 794 cm^-1^ confirms the presence of quartz admixture in the bentonite (Kumararaja et al. 2017).

After acid activation (AAB), the proton (H^+^) from the acid (HCl) penetrated the bentonite layers and attached to the hydroxyl (OH) group. Dihydroxylation and partial dissolution of the smectite structure occurred. The changes happened in the characteristic absorption bands after the acid attack in FTIR spectra are shown in Fig. 2(b). The number of OH stretching bands is the same for NB and AAB with a slight deviation of the band positions. However, the intensities of OH stretching bands at 3702 and 3619 cm^−1^ were reduced after the acid attack. The band at 3386 cm^−1^ disappeared, due to the partial dissolution of the main bentonite structure. The frequency shifts and intensity decreases in the band at 1634 cm^−1^ which was associated with adsorbed water (H–O–H stretching vibrations) compared to the NB at 1637 cm^−1^, owing to the loss of adsorbed water with the temperature used to activate the NB. The sharp vibration band near 1000 cm^−1^ is reported as stretching vibration of Si–O group; after an acid attack, the band appears with a slight shift to the higher frequency, and the intensity decreases from 63.4 to 46.2%. These results could be attributed due to the dissolution during the acid activation (Peng et al. 2005). In conclusion, the FTIR bands of ABB did not exhibit any significant change compared to the parent bentonite, indicating that the original bentonite structure was not destroyed completely. This finding was in agreement with the XRD analysis (Fig. 2(a)). Similar results were found by other authors (Morgan et al. 1985; Ravichandran 1997; Steudel et al. 2009). However, some bands show only minor shifts and decreases in the intensities. This phenomenon indicated that acid activation was more considerable for the interaction between H^+^ ions and the bentonite surface. It could also be deduced that the interaction is taking place with surface functional groups whose IR frequencies exhibit a different intensity or position. This reaction produced an amorphous, partly protonated silica phase as a final product.

#### N_2_ adsorption/desorption measurements

The textural properties of the bentonite clay before and after acid activation using N_2_ adsorption/desorption isotherms are shown in Fig. 2(c). The obtained data showed that both NB and AAB followed type IV isotherm according to IUPAC isotherm classification (Thommes et al. 2015).

Furthermore, both samples contained H_3_ hysteresis loops, which is always found on the solids consisting agglomerate particles with non-uniform size or shape of slit-shaped pores (Pawar et al. 2016). These results confirmed the isotherm for layered clay material, possibly due to the multilayer formation and capillary condensation in mesopores (pore diameter 2–50 nm) (Budsaereechai et al. 2012). The NB and AAB curves showed no significant difference, indicating that acid activation did not affect the mesoporous character of the bentonite clay sample. The obtained result in Table 2 illustrated that acid activation of bentonite significantly increased the specific surface area (from 13.19 to 29.59 m^2^/g) and total pore volume (from 0.07 to 0.09 cm^3^/g). The enhancement of textural properties of the bentonite after acid activation could be attributed to splitting of clay particles within the dissolved octahedral sheets. This enhancement could also be tracked back to open up the edges of the clay platelets due to the acid activation (Valenzuela Díaz and De Souza Santos 2001). Figure 2(d) shows the BJH pore diameter distribution patterns of the bentonite clay sample before and after the acid activation. The results revealed that acid activation decreased the pore size in the clay particles, causing an increase in the surface area. These results revealed that enhanced sorption capacity of AAB is linked to an increase in the porosity of bentonite (Toor et al. 2015).

**Table 2:** The textural properties of NB and AAB samples based on N_2_ adsorption–desorption isotherms

Properties	Samples	
	NB	AAB
BET surface area (m^2^/g)	13.19	29.59
*V*_m_ (cm^3^(STP)/g)	3.03	6.79
Total pore volume (cm^3^/g)	0.07	0.09
Mean pore diameter (nm)	22.53	11.37
Langmuir surface area (m^2^/g)	12.63	29.38
BJH surface area (m^2^/g)	15.25	27.79

#### Scanning electron microscopy and energy-dispersive X-ray studies

**Fig. 3:**
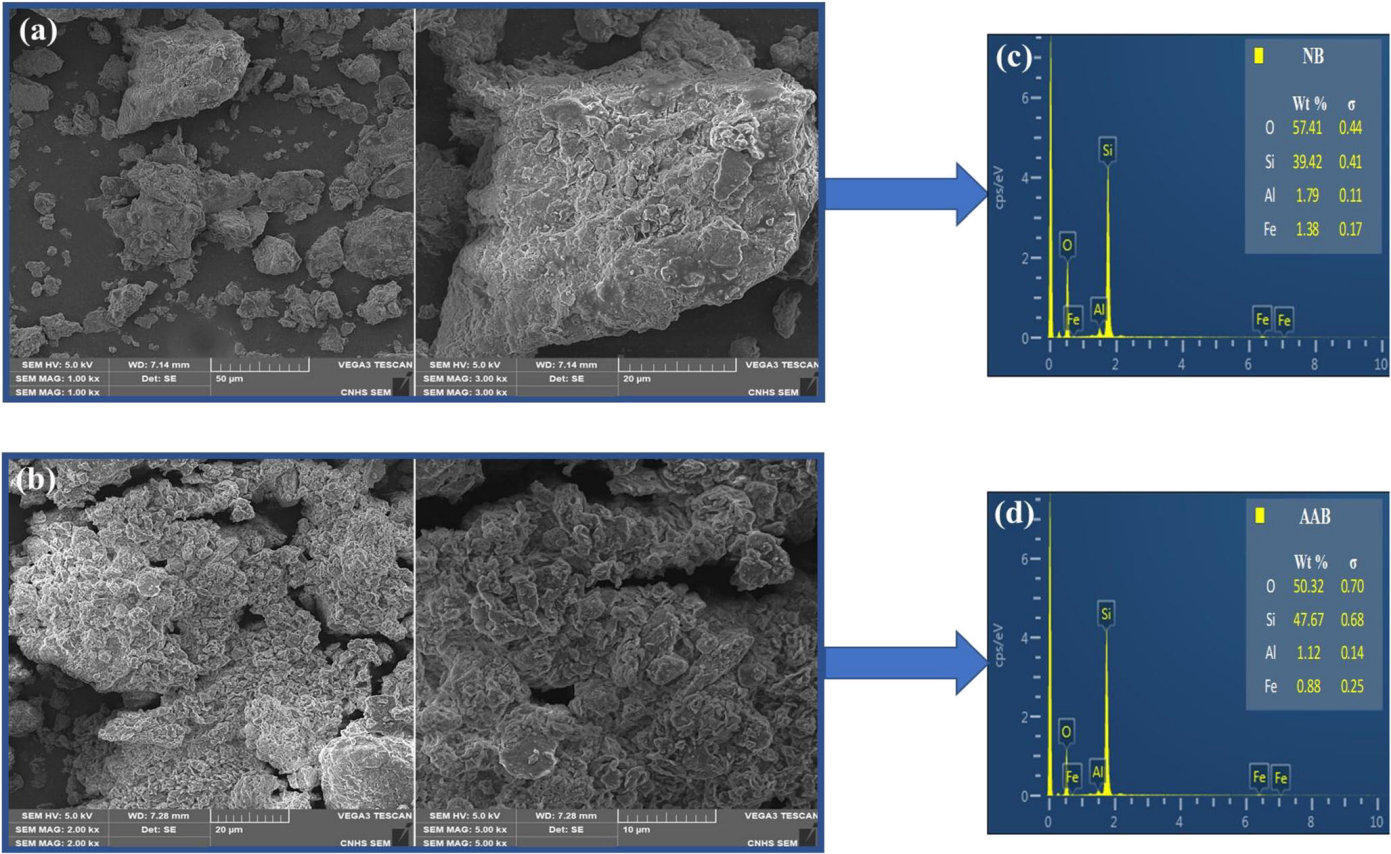
SEM images of the samples with different magnifications for (a) NB and (b) AAB, EDX spectra results for (c) NB and (d) AA

In order to investigate the effect of the acid activation on the surface morphology of bentonite, the SEM analysis was carried out for NB and AAB (Fig. 3(a, b)). The surface morphology of NB appeared with a significant difference compared to AAB sample. The surface of NB sample (Fig. 3(a)) appeared to be highly compact and irregularly shape particles. However, the surface of AAB sample (Fig. 3(b)) had larger size pores between the particles compared to the NB sample. Moreover, the AAB surface appeared to be formed by several flaky particles accumulated together in form of agglomerates. This result was found in agreement with the obtained result of BET analysis (“N_2_ adsorption/desorption measurements” section). The SEM images of AAB indicated the disaggregation and decrease in the size of the bentonite structure after the acid treatment.

Figure 3(c, d) shows the energy-dispersive X-ray (EDX) spectra of the bentonite clay sample before (NB) and after (AAB) acid activation. From the EDX results, O, Si, and Al are the most abundant constituents in the samples in which these elements are the basic elements for the smectite group. The EDX results are in agreement with the obtained analysis from XRF (Table 1). There is a variation in the bentonite composition before and after the acid attack. The HCl treatment of bentonite has caused an increase of the Si content and decrease in the content of Al and Fe, due to the partial dissolution of the octahedral sheet (Venaruzzo et al. 2002; Amari et al. 2010).

### Batch-mode sorption experiment for CIP removal

#### Effect of solution pH on CIP sorption

**Fig. 4:**
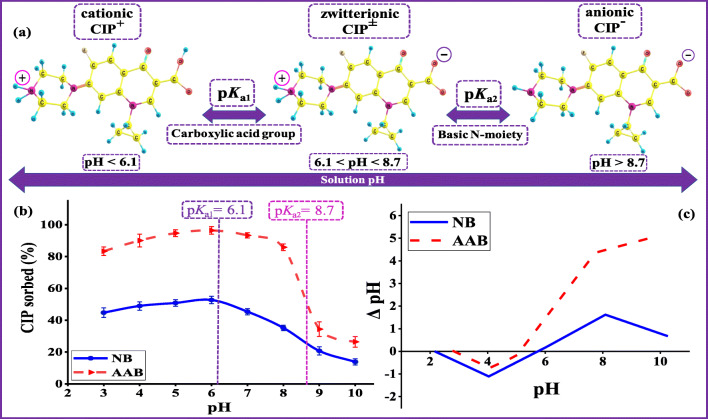
Mechanism of pH-dependent. (a) pH-dependent CIP speciation. (b) Effect of pH. (c) pH_zpc_ measurements of NB and AAB

The CIP sorption is significantly affected by the solution pH due to varied CIP speciation as well as the surface charge of the bentonite clay adsorbents. The pH_zpc_ measurements illustrate the impact of the protonation and deprotonation of the adsorbent’s surface on the sorption process. The pH_zpc_ values were found to be 6.1 and 5.0 for NB and AAB, respectively (Fig. 4). At pH < pH_zpc_, the adsorbents’ surface charge was positive, while the adsorbents surface charge was negative when pH > pH_zpc_. On the other hand, CIP molecules exist in three distinct forms as a function of the solution pH. The CIP cationic form (CIP^+^) exists when the solution pH is less than p*K*_*a*1_ (= 6.1), which attributed to protonation of the amine group (at piperazine moiety) (Fig. 4) (Wang et al. 2010). When the solution pH is higher than p*K*_*a*2_ (= 8.7), the CIP anionic form (CIP^−^) exists due to deprotonation of the amine group (piperazine moiety). At solution pH between p*K*_*a*1_ and p*K*_*a*2_, the CIP molecule becomes zwitterion (CIP^±^) (Wang et al. 2010). This behavior is due to the protonation and deprotonation of the amine group (at piperazine moiety) and carboxylic acid group, respectively (Jiang et al. 2013). Figure 4(b) shows the effect of the solution pH on CIP sorption by NB and AAB. It was observable that the CIP removal slightly increased when the solution pH was less than p*K*_*a*1_, confirming the CIP speciation (cationic form) and pH_zpc_ measurements (positive charge). Thus, the sorption mechanism of CIP mainly involves cation exchange and interlayer complexation between bentonite and CIP molecules. At pH range of 5–6.1, CIP sorption was favored due to the electrostatic attraction between negative surface charge of bentonite clay and positive charge on CIP molecule. When the solution pH (p*K*_*a*1_<pH <p*K*_*a*2_), the bentonite surface became negatively charged and the CIP molecules exist in zwitterionic form. Consequently, the CIP sorption was still high, and positively charged amine group of zwitterionic form could still contribute in the CIP sorption via cation exchange mechanism (Genç et al. 2013). Thereafter, a sharp decrease in the CIP sorption occurred, when the solution pH became higher than p*K*_*a*2_. This decrease could be explained due to the repulsion between CIP anionic form and the negatively charged bentonite clay surface. Moreover, this also confirmed that the cation exchange mechanism was no longer controlling the CIP sorption. Overall, the combined information gained from CIP speciation, pH_zpc_ measurements, and pH studies of CIP removal by NB and AAB helped to understand the sorption mechanism.

#### The effect of clay dosage on CIP sorption

**Fig. 5:**
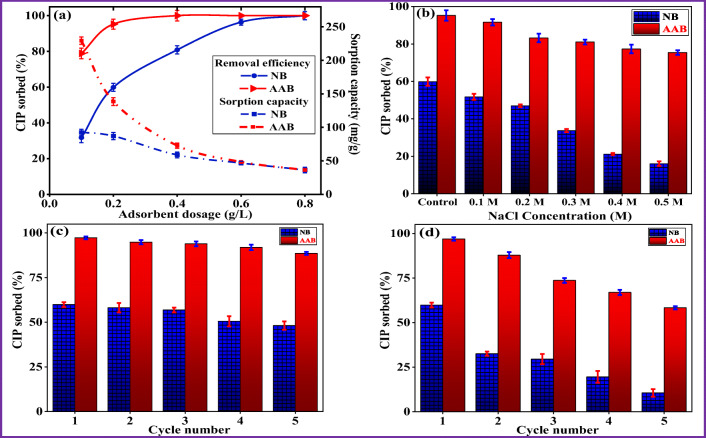
Effect of (a) adsorbent dosage and (b) ionic strength on CIP sorption onto NB and AAB, regeneration studies of CIP adsorption onto NB and AAB using (c) 0.1 M NaOH and (d) 0.1 M HCl as eluent

In order to optimize the amount of clay mass needed to reach the maximum CIP sorption, the effect of NB and AAB dosage on CIP removal efficiency and sorption capacity was assessed. The CIP sorbed (%) and sorption capacity (mg/g) versus adsorbents dosage is plotted in Fig. 5(b). The obtained results showed that the CIP removal percentage gradually increased with the increase in clay mass. This behavior could be explained due to the increase in the surface area and available binding sites. Equilibrium was reached quickly for AAB with relatively lower dose of 0.3 g/L compared to NB with sorbent dose of 0.7 g/L. This could be attributed to the acid activation of bentonite which provides more surface-active sites for sorption of CIP molecules. Based on the result, an adsorbent dose of 0.2 g/L was selected for further experiments.

#### The effect of ionic strength on CIP removal

One of the parameters affecting the removal of pharmaceutical pollutants from water is the presence of salts in aqueous solutions. Thus, the effect of ionic strength on CIP sorption was also investigated. The influence of NaCl concentrations on CIP sorption onto NB and AAB is shown in Fig. 5(b). Apparently, increasing the electrolyte concentrations inhibited the CIP removal by both adsorbents, NB and AAB. Specifically, the CIP removal dramatically decreased from 59.8% (0 M NaCl) to 16.1% (from 0 to 0.5 M NaCl) for NB, and from 95.3 to 75.5% (from 0 to 0.5 M NaCl) for AAB. It can be attributed to higher Na^+^ concentration of ions in the solution due to increasing electrolyte (NaCl) concentrations. Therefore, there is a competition between CIP cationic (CIP^+^) form (when solution pH < p*K*_*a*1_ (= 6.1)) and Na^+^ ions for the sorption sites on the bentonite surface. Similar observations were reported for CIP sorption in other studies (Akpomie et al. 2019; Xu et al. 2019).

#### CIP sorption kinetics and mechanism

**Fig. 6:**
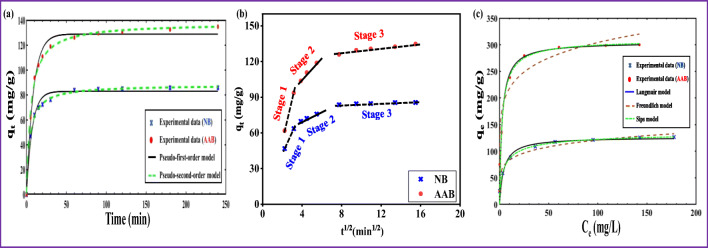
(a) Sorption kinetic modeling of CIP, (b) intra-particle diffusion model, and (c) sorption isotherms modeling of CIP (concentration: 5–200 mg/L) by NB and AAB, respectively

The effect of contact time is one of the important parameters which helps to determine the equilibration time as well as to get insight into the sorption rate and mechanism. The contact time experiments were conducted with different interaction times (5–240 min), while the other experimental conditions were maintained (CIP concentration: 30 mg/L, dose: 0.2 g/L, and pH ∼ 5.5) based on the previous batch experiment results. The contact time (min) versus sorption capacity (mg/g) for NB and AAB was plotted. The obtained results (Fig. 6(a)) revealed that the sorption capacity was dramatically enhanced in the first 30 min (due to quick sorption), attaining about 80% of the total CIP sorbed by NB and AAB. After 30 min, the sorption rate was relatively slow, and the equilibrium was reached within 120 min for NB and AAB. Therefore, a contact time of 180 min was selected for further experiments. The same sorption behavior was observed for both sorbents (NB and AAB) with different contact times; however, AAB showed about 50% higher sorption capacity. This increase in the sorption capacity could be attributed to acid activation which provided more active sites. The experimental sorption data were fitted to PFO and PSO models in order to evaluate the sorption kinetics of CIP onto NB and AAB. The computed kinetic parameters and equations for these models are listed in Table 3. The comparison of kinetic data for the two models showed that PSO could effectively describe the CIP sorption process. This observation was supported by the high *R*^2^ value, relatively low RMSE and the close match between the calculated sorption capacity (*q*_*e*, cal_) and the experimental sorption capacity (*q*_*e*, exp_) obtained from PSO for NB and AAB (Table 3). These results indicated that CIP sorption onto NB and AAB is rate-determined by chemical-sorption, where the sorption capacity is dependent on the number of active binding sites on the adsorbents (Duan et al. 2018; Wu et al. 2019).

**Table 3:** Pseudo-first-order, pseudo-second-order, and intra-particle diffusion model parameters of CIP sorption onto NB and AAB

Adsorbent	Pseudo-first-order model $\kern0.5em \left({q}_t={q}_e\left(1-{e}^{-{k}_1t}\right)\right)$				
	*k*_1_ (1/min)	*q*_e, cal_ (mg/g)	*q*_*e*, exp_ (mg/g)	RMSE	*R*^2^
NB	0.14	82.70	85.56	3.69	0.981
AAB	0.12	126.10	134.74	7.57	0.969
	Pseudo-second-order model $\left({q}_t=\frac{k_2{q}_e^2t}{1+{k}_2{q}_et}\right)$				
	*k*_2_ (g/mg min)	*q*_*e*, cal_ (mg/g)	*q*_*e*, exp_ (mg/g)	RMSE	*R*^2^
NB	0.0028	87.80	85.56	1.06	0.999
AAB	0.0014	134.70	134.74	3.31	0.994
	Intra-particle diffusion model (*q*_*t*_ = *k*_*id*_ *t*^1/2^ + *C*)				
	*k*_id_ (mg/g min^1/2^)	*C*	*q*_*e*, exp_ (mg/g)	RMSE	*R*^2^
NB	2.45	54.46	85.56	16.13	0.752
AAB	6.76	51.75	134.74	15.22	0.884

According to Weber and Morris’s theory, the sorption mechanism follows IPD when the plot curve is linear passing through the origin. Therefore, IPD becomes the main rate-controlling step. Otherwise, other mechanisms are also involved besides IPD (Bhattacharyya and Sen Gupta 2007; Wang et al. 2015). The applied experimental data to IPD kinetic model is presented in Fig. 6(b) and the calculated parameters are listed in Table 3. The IPD plots of CIP onto NB and AAB show three-linear stages. The movement of adsorbate from the bulk solution to the bentonite surface can be explained in three sequential steps: (i) surface or external diffusion, which involves the transportation of CIP molecules from sorbate to the external bentonite surface; (ii) intra-particle (internal) diffusion, which involves the transportation of CIP molecules from the external bentonite surface to the interior part of bentonite; (iii) the CIP molecules get sorbed at the active sites of bentonite (sorption) (Wu et al. 2019). Since the IDP curves of NB and AAB exhibited negative pass through the experimental data origin, this observation indicated that intra-particle diffusion is not only the rate-controlling step, but also other mechanisms also take part during the sorption process. This inference comes in agreement with the proposed mechanism of pH-dependent CIP sorption (“Effect of solution pH on CIP sorption” section).

#### CIP sorption isotherms

The equilibrium sorption isotherms of NB and AAB as a function of varied initial CIP concentration in the range of 5–200 mg/L are shown in Fig. 6(c). The obtained results showed that the sorption capacities for NB and AAB increased with increasing CIP concentrations and the saturation reached progressively. The experimental sorption data of CIP was subjected to the non-linear form of three well-known isotherms (Langmuir, Freundlich, and Sips) models, in order to get insight on the possible sorption mechanism. The calculated parameters, constants, and corresponding regression coefficients as well as the equations of each model are listed in Table 4. A careful comparison between the model’s parameters for NB and AAB revealed that Langmuir was the best fitted model with *R*^2^> 0.99 and low RMSE values. Moreover, the calculated maximum sorption capacity (*q*_max_) of Langmuir model and the experimental data values were found in good agreement with the calculated (from model) values, confirming the validity of this model to describe the CIP sorption process. The Langmuir isotherm also suggested that sorption takes place at particular homogenous sites within adsorbent surface, which is restricted to the formation of a monolayer sorption due to a strong interaction between the adsorbent and the adsorbate (Rusmin et al. 2015). Furthermore, favorability of the sorption process based on Langmuir model was evaluated by the dimensionless equilibrium parameter (*R*_*L*_). *R*_*L*_ values were in the range between 0 and 1 for NB and AAB, indicating a favorable sorption process and strong binding between CIP molecules and the bentonite adsorbent (Genç et al. 2013). The AAB exhibited maximum monolayer sorption capacity of 305.20 mg/g compared to 126.56 mg/g in case of NB, indicating that acid activation of bentonite enhanced the CIP sorption by twofold, compared to NB.

**Table 4:** Langmuir, Freundlich, and Sips isotherm model parameters for CIP sorption onto NB and AAB

Adsorbent	Langmuir isotherm model $\left({q}_e=\frac{q_m{K}_L{C}_e}{1+{K}_L{C}_e}\right)$					
	*q*_max(cal)_ (mg/g)	*q*_max(exp)_(mg/g)	*K*_*L*_ (dm^3^/mg)	*R*_*L*_	*R*^2^	RMSE
NB	126.56	126.53	0.24	0.02	0.990	3.29
AAB	305.20	300.03	0.39	0.01	0.987	28.56
	Freundlich isotherm model $\left({q}_e={K}_F{C}_e^{1/n\operatorname{}}\right)$					
	*K*_*F*_ (mg/g) (L/mg)^1/n^		n	*R*^2^		RMSE
NB	57.37		6.16	0.966		5.97
AAB	186.32		9.91	0.926		38.41
	Sips isotherm model $\left({q}_e=\frac{K_s{C}_e^{\beta s}}{1+{a}_s{C}_e^{\beta s}}\right)$					
	*K*_*S*_ (L/g)	*β*_*S*_	a_*S*_	*R*^2^		RMSE
NB	47.70	0.68	0.35	0.934		11.22
AAB	133.60	0.90	0.43	0.928		35.48

A comparison of obtained maximum monolayer sorption capacity (*q*_max_) of NB and AAB along with *q*_max_ of several adsorbents, reported in the literature is listed in Table 5. The carbonaceous adsorbents showed fairly high *q*_max_ compared to the other adsorbents. This could be attributed to differences in the adsorbent properties including specific surface area and pore size as well as the interactions between CIP and adsorbents and other experimental factors. However, preparing and utilizing the carbonaceous adsorbents are relatively expensive compared to the non-carbonaceous adsorbents, due to the consumption of high amount of energy. Among the non-carbonaceous adsorbents, natural and activated bentonite exhibited high CIP sorption capacity especially AAB. This behavior could be ascribed to the unique porous properties, the presence of functional groups, considerable surface area, and swelling properties. Therefore, acid activation of bentonite could be considered as one of the excellent bentonite modification techniques, which significantly enhanced the sorption capacity of CIP. The wide availability of bentonite, simple modification method, and most likely low operating costs show that modified bentonite is a very suitable sorbent in water treatment for the removal of ciprofloxacin.

**Table 5:** Comparison of maximum adsorption capacities of various adsorbents reported in the literature for CIP sorption

Adsorbents	Adsorption capacity *q*_max_ (mg/g)	Adsorbent dosage (g/L)	References
Jerivá activated carbon (40 °C)	335.80	1.5	(de Oliveira et al. 2019)
Modified flax noil cellulose (cationic)	238.70	0.1	(Hu and Wang 2016)
Synthesized layered chalcogenide (25 °C)	230.9	0.1	(Li et al. 2015)
Rectorite	135	10.0	(Wang et al. 2011)
Biochar (rice straw)	131.58	0.4	(Zeng et al. 2018)
Graphene oxide/calcium alginate (6%)	66.3	2.0	(Wu et al. 2013)
Synthesized Nanoceria	49.38	0.2	(Rahdar et al. 2019)
Bamboo charcoal	36.0	4.0	(Wang et al. 2017)
Illite	33	10.0	(Wang et al. 2011)
Halloysite	21.7	1.0	(Duan et al. 2018)
Activated red mud	19.1	5.0	(Balarak et al. 2017)
Schorl	8.5	2.0	(Yin et al. 2018)
Kaolinite	6.3	100.0	(Li et al. 2011)
Natural bentonite	126.6	0.2	Present study
Acid activated bentonite	305.2	0.2	Present study

#### Regeneration studies of NB and AAB

The recyclability of adsorbents has great importance to make the process cost-effective in water treatment. The regenerated NB and AAB adsorbents were treated for five cycles by repeating the adsorption/desorption process with a mild acid and base solution as the desorbing agent. The obtained results showed that NaOH (0.1 M) was better eluent than HCl (0.1 M) for the regeneration of NB and AAB (Fig. 5(c, d)). The sorption of CIP (in case of NaOH) decreased from 59.9 to 48.1% for NB and slightly decreased from 97.3 to 88.5% for AAB after five consecutive cycles (Fig. 5(c)). The decrease in CIP sorption could be attributed to the loss of adsorbent mass and active sorption sites during eluent washing. On the other hand, the removal efficiency of CIP decreased dramatically from 59.8 to 10.6 % for NB, and significantly from 96.9 to 58.3 % for AAB from the 1st to 5th cycle with HCl as eluent (Fig. 5(d)). The experimental results revealed that AAB can be successfully regenerated by NaOH treatment using the adsorption/desorption process.

### Continuous-mode fixed-bed column studies

**Fig. 7:**
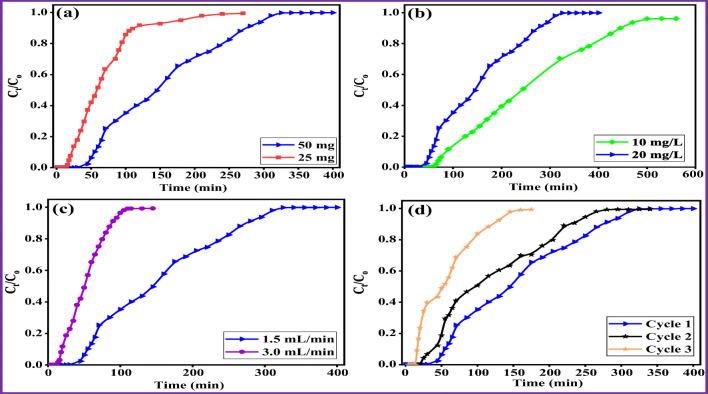
Effect of various parameters on breakthrough curve of CIP sorption in the fixed-bed column onto AAB: (a) adsorbent loading 50 and 25 mg of AAB, (b) CIP concentrations 10 and 20 mg/L, (c) flow rate 1.5 and 3 mL/min, and (d) column regeneration

The batch experiments revealed excellent sorption capacity of AAB for removal of CIP from aqueous solution. Therefore, AAB adsorbent was utilized in the continuous-mode fixed-bed column studies, to scale up the process. Various parameters such as AAB loading, inlet CIP concentration, and flow rate were examined in order to optimize and get insight into the feasibility and cost-effectiveness of the removal process. The effect of aforementioned parameters on CIP removal efficiency is presented in Fig. 7. The calculated parameters for each column are listed in Table 6.

**Table 6:** The effect of flow rate, adsorbent loading, and initial CIP concentration on the total adsorbed CIP (*q*_total_), equilibrium uptake (*q*_eq_), total removal efficiency of the column (*RE %*), and total unadsorbed CIP concentration at equilibrium (*C*_eq_)

Experiments	Parameters										
	*C*_*o*_ (mg/L)	*Q* (mL/min)	*M*_AAB_ (mg)	*V*_eff_ (mL)	*t*_*b*_ (min)	*t*_total_ (min)	*q*_total_ (mg)	*q*_bed_ (mg/g)	*m*_total_ (mg)	*RE* (%)	*C*_eq_ (mg/L)
Column 1	20	1.50	50	600	40	400	4.86	97.12	12.78	37.99	13.21
Column 2	20	1.50	25	405	16	270	2.18	87.12	8.63	25.25	15.92
Column 3	10	1.50	50	840	60	560	4.11	82.13	8.95	45.89	5.77
Column 4	20	3.00	50	435	14	145	3.34	66.81	9.27	36.05	13.62
Column regeneration											
Cycle 1	20	1.50	50	600	40	400	4.86	97.12	12.78	37.99	13.21
Cycle 2	20	1.50	50	465	19	310	3.09	61.85	9.91	31.22	14.65
Cycle 3	20	1.50	50	217.50	11	145	1.41	28.14	4.63	30.37	14.83
Total	20	1.50	50	1282	70	855	9.36	187.11	27.32	–	–

#### Effect of AAB loading

The column experiments were conducted by two different AAB adsorbent loadings (50 and 25 mg), to evaluate the effect of the adsorbent loading on the performance of the breakthrough curve and time (Fig. 7(a)). During the experiment, the other parameters were kept constant (pH~5.5, initial CIP concentration = 20 mg/L, and flow rate = 1.5 mL/min). The obtained data showed that the breakthrough time and steepness of the breakthrough curve were influenced by the adsorbent loadings. By increasing the adsorbent loading (*M*_AAB_) from 25 to 50 mg, the column removal efficiency (*RE* %) for CIP increased from 25.25 to 37.99%, and corresponding increase in the CIP adsorbed quantity (*q*_total_) by the column system from 2.18 to 4.86 mg. The breakthrough time was also increased from 16 to 40 min by increasing the adsorbent loading. These results revealed that increasing AAB amount enhanced the performance of column and increased the total CIP removal efficiency. Furthermore, the exhaustion time also increased significantly from 270 to 400 min with increasing AAB loading from 25 to 50 mg. This behavior could be attributed to increasing the bed height due to increased AAB amount, which provided more available sorption sites and enough residence time for CIP solution inside the bed to interact with the adsorbent in the column system (Baral et al. 2009).

#### Effect of inlet CIP concentration

In order to determine the influence of inlet CIP concentration on the breakthrough curve and breakthrough time, the column experiments using AAB were conducted with two different initial CIP concentrations of 10 and 20 mg/L (Fig. 7(b)). In this experiment, the other parameters (pH~5.5, AAB loading = 50 mg, and flow rate = 1.5 mL/min) were kept constant. The obtained results showed that increasing the inlet CIP concentrations (*C*_*o*_) from 10 to 20 mg/L significantly affected the CIP removal efficiency of AAB. The results demonstrated a decrease in the column removal efficiency from 45.89 to 37.99% when *C*_*o*_ increased from 10 to 20 mg/L, respectively (Table 6). Similarly, increasing CIP concentration influenced the breakthrough time, which was decreased from 60 to 40 min and also the total column operational time from 560 to 400 min. Furthermore, the column with the highest *C*_*o*_ exhibited steeper slope curve and quickly reached the exhaustion time comparing to the column with the lowest *C*_*o*_. This behavior can be explained as the available binding sites in the adsorbent became rapidly saturated with increasing the inlet concentration. On the other hand, the total CIP passed through the column system (*m*_total_) and uptake capacity (*q*_bed_) were found to be increased from 8.95 to 12.78 mg and from 82.13 to 97.12 mg/g, respectively. This increase in uptake capacity could be due to high inlet CIP concentration, providing a higher driving force for the transfer process to overcome the AAB mass transfer resistance (Baral et al. 2009). These observations are in agreement with those found by other researchers (Golie and Upadhyayula 2016; Ahmed and Hameed 2018).

#### Effect of flow rate

Flow rate is considered as one of the important factors which affects the performance of continuous-mode sorption system. The influence of flow rate on the breakthrough curve and time was studied by two different flow rates (1.5 and 3.0 mL/min), whereas the other parameters (pH~5.5, AAB loading = 50 mg, and initial CIP concentration = 20 mg/L) were kept constant. Figure 7(c) shows that increasing the flow rate from 1.5 to 3.0 mL/min had a negative impact on the breakthrough time and breakthrough curve, which resulted in shorter breakthrough time (from 40 to 14 min) with sharp breakthrough curve shape. The total column operational time was also significantly decreased from 400 to 145 min with increasing the flow rate from 1.5 to 3.0 mL/min. Table 6 illustrates that the higher flow rate also adversely affected the removal efficiency of AAB as *RE* % decreased from 37.99 to 36.05%, *q*_bed_ from 97.12 to 66.81 mg/g and *q*_total_ from 4.86 to 3.34 mg. This negative impact with the higher flow rate could be explained due to the insufficient residence time (contact time) of the adsorbate molecule with the adsorbent. Therefore, the adsorbate molecule passed through AAB inside the column before the equilibrium state was reached, which led to incomplete use of the adsorbent´s full capacity (Daneshvar et al. 2019). In contrast, utilizing lowest flow rate provides sufficient residence time to the CIP molecule to be in contact with the adsorbent in continuous sorption system (Maged et al. 2019). These findings are in agreement with a previous study of TC sorption from water using Fe/graphene (Alatalo et al. 2019).

#### Column regeneration

The column regeneration experiments were conducted to get insight onto the feasibility of reusing AAB adsorbent in the continuous-mode fixed-bed column. From aforementioned experiments, the highest value of maximum sorption capacity was found to be 97.12 mg/g with column 1, which was used in further regeneration experiment. The selected column for the experiment was performed under the optimum experimental conditions of pH~5.5, AAB loading = 50 mg, initial CIP concentration = 20 mg/L, and flow rate = 1.5 mL/min. For the first cycle, the AAB adsorbent was saturated after 400 min with maximum sorption capacity of 97.12 mg/g as shown in Table 6 and Fig. 7(d). The second cycle showed slight decrease in the saturation time of 310 min, while the removal efficiency also decreased from 37.99 to 31.22%. After the third cycle, the saturation was reached quickly (145 min) with a sorption capacity of 28.14 mg/g. The reusability of AAB for three sequential cycles successfully enhanced the overall sorption capacity and increased the total amount of treated CIP solutions.

### Sorption mechanism of CIP onto AAB

**Fig. 8:**
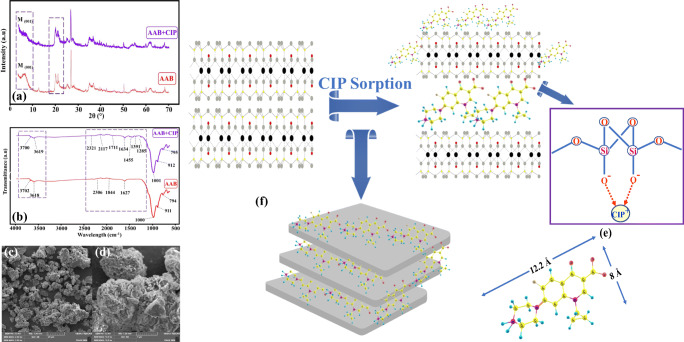
(a) XRD analysis of AAB before and after CIP adsorption, (b) FTIR spectra of AAB before and after CIP adsorption, SEM image of AAB (c) before and (d) after CIP adsorption, (e) dimension of the CIP molecule, and (f) possible sorption mechanism of CIP adsorption onto AAB

The CIP sorption mechanism onto AAB was investigated by combining the XRD, FTIR, and SEM results before and after CIP sorption. The comparison between XRD patterns before and after CIP sorption is shown in Fig. 8(a). The *d*_*001*_ spacing of the montmorillonite peak (AAB) at 2*θ =* 5.6° (15.87 Å) was expanded after CIP sorption to 17.07 Å at 2*θ =* 5.02°. The dimension of the CIP molecule is 12.2 Å length, 8.0 Å height, and 4.1 Å thickness (Fig. 8(e)) (Turel and Golobic 2003). The expanding of the montmorillonite peak after CIP sorption confirmed that the CIP molecules were successfully intercalated in the interlayer structure of AAB. Furthermore, the intensity of the montmorillonite peaks at 2*θ =* 5.02° and 20° was decreased. This behavior could be attributed to the fact that CIP molecules strongly bonded with hydroxyl groups of AAB. Similar findings were reported by other authors in the adsorption of pharmaceutical compounds (Kulshrestha et al. 2004; Wang et al. 2010). The comparison between FTIR spectra before and after CIP sorption is shown in Fig. 8(b). Originally, the main characteristic bands of pure CIP appear in the range of 1250–1850 cm^−1^ (Wang et al. 2010). This range includes three important stretching vibration bands of C*=*O for the carboxylic acid, C*=*O for ketone, and C–O stretching (carboxylic acid) coupling with O–H deformation vibration at wavelengths 1707, 1624, and 1274 cm^−1^, respectively (Trivedi and Vasudevan 2007). In addition, the band at 1385 cm^−1^ attributed to the protonation of the amine group of the piperazine moiety (Gu and Karthikeyan 2005). On contrary, bentonite clays in the range between 3000 and 1300 cm^−1^ are free from vibration bands, except the water bending vibration around 1630 cm^−1^, which is an important band in sorption of CIP molecules (Chang et al. 2009). After CIP sorption under the previously optimized conditions, the main changes appeared in the range between 2300 and 1200 cm^−1^, indicating that CIP molecules were successfully sorbed into AAB. Two significant changes in the FTIR spectra were observed. First, the band at 1627 cm^−1^ was shifted to higher wavelength at 1634 cm^−1^. This shift could be attributed to the formation of hydrogen bonding between released carboxylic acid groups and basal oxygen atoms of montmorillonite. Similar observation was found by other authors when studied CIP spectroscopic investigation speciation on goethite (Trivedi and Vasudevan 2007). Second, the appearance of new bands at 1285, 1391, and 1455 cm^−1^ suggested the formation of a new bond or electrostatic attraction between negatively charged AAB surface and protonated amine group of CIP. Figure 8(c, d) shows SEM images before and after CIP sorption, respectively. The obtained result from SEM showed that the AAB surface lost the roughness and became more stacked. This behavior could be attributed to the CIP molecules filling the adsorbent pores after sorption. Overall, this study suggests that the sorption of CIP onto AAB involves four proposed mechanisms (Fig. 8(f)): (i) intercalation of CIP in the interlayer space of AAB via cation exchange (confirmed by XRD analysis), (ii) hydrogen bonding between basal oxygen atoms of AAB and carboxylic acid of CIP molecules (confirmed by FTIR analysis), (iii) electrostatic interaction between the negative binding sites on AAB surface and cationic species of CIP (confirmed by FTIR analysis and pH studies), and (iv) surface complexation and pore filling (confirmed by SEM analysis).

## Conclusions

Natural bentonite clay, collected from Egypt, was studied to evaluate its sorption potential to remove ciprofloxacin antibiotics from water. Mild pre-treatment and acid activation technique were opted to enhance the adsorptive efficiency of the bentonite clay towards CIP removal. The maximum sorption capacity after acid activation successfully increased from 126.56 to 305.20 mg/g under the optimized conditions. Both NB and AAB were extensively characterized to determine the changes in the adsorbent before and after acid activation. The calculated Al_2_O_3_/SiO_2_ ratio, given by XRF analysis, indicated that both NB and AAB consisted mostly of montmorillonite. The XRD and FTIR results revealed that acid activation of bentonite did not damage the inherent layered structure of the natural bentonite. The pH-dependent study confirmed that cation exchange and interlayer complexation between bentonite and CIP molecules were the main sorption mechanism, while kinetic studies exhibited that PSO was the best model to describe CIP sorption process and also chemisorption was the responsible mechanism for CIP removal. Using the characterization techniques for AAB before and after CIP sorption, several sorption mechanisms were proposed and confirmed, i.e., intercalation of CIP in the interlayer space, electrostatic interaction, hydrogen bonding between basal oxygen atoms and carboxylic acid of CIP, and surface complexation and pore filling. Mild base solution (0.1 M NaOH) showed the ability to reuse the adsorbents for 5 cycles without significant loss in sorption capacity. Furthermore, the maximum sorption capacity in the continuous flow mode was found to be 97.12 mg/g. In addition, the regeneration of AAB in column system was achieved successfully for three sequential cycles with total sorption capacity of 178.11 mg/g. The low-cost recyclability of AAB certainly would simplify its practical applications in water treatment.

## Electronic supplementary material

ESM 1 (DOCX 49 kb)

## Nomenclature

*θ*: The angle of diffraction (°)
*q*_*m*_: The Langmuir constant associated with sorption capacity (mg/g)
*d*: The basal spacing (Å)
*K*_*L*_: The constants of Langmuir (L/mg)
*n*: The path differences between the reflected waves which equals an integral number of wavelengths (*λ*)
*K*_*F*_: The constants of Freundlich (mg/g) (L/mg)^1/n^
*q*_*e*_: The sorption capacity of the adsorbent (mg/g)
*n*: The exponent of Freundlich related to sorption intensity
*C*_*i*_: The initial CIP concentration (mg/L)
*K*_*s*_: The Sips constant (L/mg)
*C*_*e*_: The equilibrium CIP concentration (mg/L)
*R*^2^: The correlation coefficient
W: The mass of clay adsorbent (g)
RMSE: The root mean square error
*V*: The volume of CIP solution (L)
*Q*: The flow rate (mL/min)
*q*_*t*_: The adsorption capacity (mg/g) at time t (min)
*t*_total_: The total flow time (min)
*k*_1_: The constants of PFO (1/min)
*C*_ad_: The adsorbed CIP concentration (mg/L)
*k*_2_: The constants of PSO (g/mg min)
*m*: The AAB dry mass (g)
*k*_*id*_: The constants of IPD (mg/g min^1/2^)
IUPAC: The International Union of Pure and Applied Chemistry

## References

[CR1] Abdou MI, Al-sabagh AM, Dardir MM (2013). Evaluation of Egyptian bentonite and nano-bentonite as drilling mud. Egypt J Pet.

[CR2] Abu-Danso E, Peräniemi S, Leiviskä T, Kim TY, Tripathi KM, Bhatnagar A (2020). Synthesis of clay-cellulose biocomposite for the removal of toxic metal ions from aqueous medium. J Hazard Mater.

[CR3] Ahmed MJ, Hameed BH (2018). Removal of emerging pharmaceutical contaminants by adsorption in a fixed-bed column: a review. Ecotoxicol Environ Saf.

[CR4] Akpomie KG, Fayomi OM, Ezeofor CC, Sha’Ato R, van Zyl WE (2019). Insights into the use of metal complexes of thiourea derivatives as highly efficient adsorbents for ciprofloxacin from contaminated water. Trans R Soc South Africa.

[CR5] Alatalo S-M, Daneshvar E, Kinnunen N, Meščeriakovas A, Thangaraj SK, Jänis J, Tsang DCW, Bhatnagar A, Lähde A (2019). Mechanistic insight into efficient removal of tetracycline from water by Fe/graphene. Chem Eng J.

[CR6] Amari A, Chlendi M, Gannouni A, Bellagi A (2010). Optimised activation of bentonite for toluene adsorption. Appl Clay Sci.

[CR7] Bagheri M, Al-jabery K, Wunsch D, Burken JG (2020). Examining plant uptake and translocation of emerging contaminants using machine learning: implications to food security. Sci Total Environ.

[CR8] Balarak D, Kord Mostafapour F, Joghataei A (2017). Environmental communication kinetics and mechanism of red mud in adsorption of ciprofl oxacin in aqueous solution. J NAAS J Score.

[CR9] Baral SS, Das N, Ramulu TS, Sahoo SK, Das SN, Chaudhury GR (2009). Removal of Cr(VI) by thermally activated weed Salvinia cucullata in a fixed-bed column. J Hazard Mater.

[CR10] Bhandari A, Close LI, Kim W, Hunter RP, Koch DE, Surampalli RY (2008). Occurrence of ciprofloxacin, sulfamethoxazole, and azithromycin in municipal wastewater treatment plants. Pract Period Hazardous, Toxic, Radioact Waste Manag.

[CR11] Bhattacharyya KG, Sen Gupta S (2007). Influence of acid activation of kaolinite and montmorillonite on adsorptive removal of Cd(II) from water. Ind Eng Chem Res.

[CR12] Budsaereechai S, Kamwialisak K, Ngernyen Y (2012) Adsorption of lead, cadmium and copper on natural and acid activated bentonite clay. Asia Pac J Sci Technol 17(5):800–810

[CR13] Carabineiro SAC, Thavorn-Amornsri T, Pereira MFR, Figueiredo JL (2011). Adsorption of ciprofloxacin on surface-modified carbon materials. Water Res.

[CR14] Carneiro RB, Sabatini CA, Santos-Neto ÁJ, Zaiat M (2019). Feasibility of anaerobic packed and structured-bed reactors for sulfamethoxazole and ciprofloxacin removal from domestic sewage. Sci Total Environ.

[CR15] Chang P-H, Li Z, Jiang W-T, Jean J-S (2009). Adsorption and intercalation of tetracycline by swelling clay minerals. Appl Clay Sci.

[CR16] Chen H, Gao B, Li H (2015). Removal of sulfamethoxazole and ciprofloxacin from aqueous solutions by graphene oxide. J Hazard Mater.

[CR17] Chen S, Yue Q, Gao B, Li Q, Xu X, Fu K (2012). Adsorption of hexavalent chromium from aqueous solution by modified corn stalk: a fixed-bed column study. Bioresour Technol.

[CR18] Daneshvar E, Zarrinmehr MJ, Kousha M et al (2019) Hexavalent chromium removal from water by microalgal-based materials: adsorption, desorption and recovery studies. Bioresour Technol 293. 10.1016/j.biortech.2019.122064 10.1016/j.biortech.2019.122064 31491650

[CR19] de Oliveira CC, Costa Rodrigues DL, Lima ÉC (2019). Kinetic, equilibrium, and thermodynamic studies on the adsorption of ciprofloxacin by activated carbon produced from Jerivá (Syagrus romanzoffiana). Environ Sci Pollut Res.

[CR20] Diwan V, Tamhankar AJ, Khandal RK, Sen S, Aggarwal M, Marothi Y, Iyer RV, Sundblad-Tonderski K, Stålsby- Lundborg C (2010) Antibiotics and antibiotic-resistant bacteria in waters associated with a hospital in Ujjain, India. BMC Public Health 10. 10.1186/1471-2458-10-414 10.1186/1471-2458-10-414 PMC2912816 20626873

[CR21] Djomgoue P, Njopwouo D (2013) FT-IR spectroscopy applied for surface clays characterization*. J Surf Eng Mater Adv Technol:275–282. 10.4236/jsemat.2013.34037

[CR22] Dorival-García N, Zafra-Gómez A, Navalón A, González J, Vílchez JL (2013). Removal of quinolone antibiotics from wastewaters by sorption and biological degradation in laboratory-scale membrane bioreactors. Sci Total Environ.

[CR23] Duan W, Wang N, Xiao W, Zhao Y, Zheng Y (2018). Ciprofloxacin adsorption onto different micro-structured tourmaline, halloysite and biotite. J Mol Liq.

[CR24] Emmerich K, Madsen FT, Kahr G (1999) Dehydroxylation behavior of heat-treated and steam-treated homoionic cis-vacant montmorillonites. Clay Clay Miner 47(5):591–604

[CR25] Falaras P, Kovanis I, Lezou F, Seiragakis G (1999). Cottonseed oil bleaching by acid-activated montmorillonite. Clay Miner.

[CR26] Felycia SI, Soetaredjo E, Ayucitra A (2015) Clay materials for environmental remediation. Springer International Publishing, Berlin

[CR27] Freundlich H (1924). Kolloidchemie und Biologie. Naturwissenschaften.

[CR28] Gates WP (2006). Chapter 12.3 X-ray absorption spectroscopy. Dev Clay Sci.

[CR29] Genç N, Dogan EC (2015). Adsorption kinetics of the antibiotic ciprofloxacin on bentonite, activated carbon, zeolite, and pumice. Desalin Water Treat.

[CR30] Genç N, Dogan EC, Yurtsever M (2013). Bentonite for ciprofloxacin removal from aqueous solution. Water Sci Technol.

[CR31] Golie WM, Upadhyayula S (2016). Continuous fixed-bed column study for the removal of nitrate from water using chitosan/alumina composite. J Water Process Eng.

[CR32] Gu C, Karthikeyan KG (2005). Sorption of the antimicrobial ciprofloxacin to aluminum and iron hydrous oxides. Environ Sci Technol.

[CR33] Ho Y, McKay G (1999). Pseudo-second order model for sorption processes. Process Biochem.

[CR34] Holmboe M, Wold S, Jonsson M (2012). Porosity investigation of compacted bentonite using XRD profile modeling. J Contam Hydrol.

[CR35] Homem V, Santos L (2011) Degradation and removal methods of antibiotics from aqueous matrices - a review. J Environ Manage 92:2304–2347 10.1016/j.jenvman.2011.05.023 21680081

[CR36] Hu D, Wang L (2016). Adsorption of ciprofloxacin from aqueous solutions onto cationic and anionic flax noil cellulose. Desalin Water Treat.

[CR37] Jiang WT, Chang PH, Wang YS, Tsai Y, Jean JS, Li Z, Krukowski K (2013). Removal of ciprofloxacin from water by birnessite. J Hazard Mater.

[CR38] Kulshrestha P, Giese RF, Aga DS (2004). Investigating the molecular interactions of oxytetracycline in clay and organic matter: insights on factors affecting its mobility in soil. Environ Sci Technol.

[CR39] Kumararaja P, Manjaiah KM, Datta SC, Sarkar B (2017). Remediation of metal contaminated soil by aluminium pillared bentonite: synthesis, characterisation, equilibrium study and plant growth experiment. Appl Clay Sci.

[CR40] Lagergren (1898). About the theory of so-called adsorption of soluble substances. Sven Vetenskapsakad Handingarl.

[CR41] Langmuir I (1918). The adsorption of gases on plane surfaces of glass, mica and platinum. J Am Chem Soc.

[CR42] Larsson DGJ, de Pedro C, Paxeus N (2007). Effluent from drug manufactures contains extremely high levels of pharmaceuticals. J Hazard Mater.

[CR43] Li B, Zhang T (2012). pH significantly affects removal of trace antibiotics in chlorination of municipal wastewater. Water Res.

[CR44] Li L, Zou D, Xiao Z, Zeng X, Zhang L, Jiang L, Wang A, Ge D, Zhang G, Liu F (2019). Biochar as a sorbent for emerging contaminants enables improvements in waste management and sustainable resource use. J Clean Prod.

[CR45] Li X, Wang W, Dou J, Gao J, Chen S, Quan X, Zhao H (2016). Dynamic adsorption of ciprofloxacin on carbon nanofibers: quantitative measurement by in situ fluorescence. J Water Process Eng.

[CR46] Li JR, Wang YX, Wang X, Yuan B, Fu ML (2015). Intercalation and adsorption of ciprofloxacin by layered chalcogenides and kinetics study. J Colloid Interface Sci.

[CR47] Li Z, Hong H, Liao L, Ackley CJ, Schulz LA, MacDonald RA, Mihelich AL, Emard SM (2011). A mechanistic study of ciprofloxacin removal by kaolinite. Colloids Surf B: Biointerfaces.

[CR48] Maged A, Iqbal J, Kharbish S, Ismael IS, Bhatnagar A (2020). Tuning tetracycline removal from aqueous solution onto activated 2:1 layered clay mineral: Characterization, sorption and mechanistic studies. J Hazard Mater.

[CR49] Maged A, Ismael IS, Kharbish S, Sarkar B, Peräniemi S, Bhatnagar A (2019). Enhanced interlayer trapping of Pb(II) ions within kaolinite layers: intercalation, characterization, and sorption studies. Environ Sci Pollut Res.

[CR50] Martins AF, Vasconcelos TG, Henriques DM, Frank CS, König A, Kümmerer K (2008). Concentration of ciprofloxacin in brazilian hospital effluent and preliminary risk assessment: a case study. CLEAN – Soil, Air, Water.

[CR51] Morgan DA, Shaw DB, Sidebottom MJ, Soon TC, Taylor RS (1985). The function of bleaching earths in the processing of palm, palm kernel and coconut oils. J Am Oil Chem Soc.

[CR52] Nasuhoglu D, Rodayan A, Berk D, Yargeau V (2012). Removal of the antibiotic levofloxacin (LEVO) in water by ozonation and TiO2 photocatalysis. Chem Eng J.

[CR53] Noyan H, Önal M, Sarıkaya Y (2007). The effect of sulphuric acid activation on the crystallinity, surface area, porosity, surface acidity, and bleaching power of a bentonite. Food Chem.

[CR54] Pawar RR, Lalhmunsiama BHC, Lee SM (2016). Activated bentonite as a low-cost adsorbent for the removal of Cu(II) and Pb(II) from aqueous solutions: batch and column studies. J Ind Eng Chem.

[CR55] Peng X, Luan Z, Chen F, Tian B, Jia Z (2005). Adsorption of humic acid onto pillared bentonite. Desalination.

[CR56] Petit S (2006). Chapter 12.6 Fourier transform infrared spectroscopy. Dev Clay Sci.

[CR57] Rahdar A, Rahdar S, Ahmadi S, Fu J (2019). Adsorption of ciprofloxacin from aqueous environment by using synthesized nanoceria. Ecol Chem Eng S.

[CR58] Rakshit S, Sarkar D, Elzinga EJ, Punamiya P, Datta R (2013). Mechanisms of ciprofloxacin removal by nano-sized magnetite. J Hazard Mater.

[CR59] Ravichandran J (1997). Properties and catalytic activity of acid-modified montmorillonite and vermiculite. Clay Clay Miner.

[CR60] Rodriguez-Narvaez OM, Peralta-Hernandez JM, Goonetilleke A, Bandala ER (2017). Treatment technologies for emerging contaminants in water: a review. Chem Eng J.

[CR61] Rusmin R, Sarkar B, Liu Y, McClure S, Naidu R (2015). Structural evolution of chitosan-palygorskite composites and removal of aqueous lead by composite beads. Appl Surf Sci.

[CR62] Sips R (1948). On the structure of a catalyst surface. J Chem Phys.

[CR63] Srinivasan R (2011). Advances in application of natural clay and its composites in removal of biological, organic, and inorganic contaminants from drinking water. Adv Mater Sci Eng.

[CR64] Steudel A, Batenburg LF, Fischer HR, Weidler PG, Emmerich K (2009). Alteration of swelling clay minerals by acid activation. Appl Clay Sci.

[CR65] Sun S-P, Guo H-Q, Ke Q et al (2009) Degradation of antibiotic ciprofloxacin hydrochloride by photo-Fenton oxidation process. Environ Eng Sci 26:753–759. 10.1089/ees.2008.0076

[CR66] Thommes M, Kaneko K, Neimark AV, Olivier JP, Rodriguez-Reinoso F, Rouquerol J, Sing KSW (2015). Physisorption of gases, with special reference to the evaluation of surface area and pore size distribution (IUPAC Technical Report). Pure Appl Chem.

[CR67] Toor M, Jin B, Dai S, Vimonses V (2015). Activating natural bentonite as a cost-effective adsorbent for removal of Congo-red in wastewater. J Ind Eng Chem.

[CR68] Trivedi P, Vasudevan D (2007). Spectroscopic investigation of ciprofloxacin speciation at the goethite−water interface. Environ Sci Technol.

[CR69] Turel I, Golobic A (2003). Crystal structure of ciprofloxacin hydrochloride 1.34-hydrate. Anal Sci.

[CR70] Uddin MK (2017). A review on the adsorption of heavy metals by clay minerals, with special focus on the past decade. Chem Eng J.

[CR71] Valenzuela Díaz FR, De Souza SP (2001). Studies on the acid activation of Brazilian smectitic clays. Quim Nova.

[CR72] Vasquez MI, Hapeshi E, Fatta-Kassinos D, Kümmerer K (2013). Biodegradation potential of ofloxacin and its resulting transformation products during photolytic and photocatalytic treatment. Environ Sci Pollut Res.

[CR73] Venaruzzo J, Volzone C, Rueda M, Ortiga J (2002). Modified bentonitic clay minerals as adsorbents of CO, CO2 and SO2 gases. Microporous Mesoporous Mater.

[CR74] Wang CJ, Li Z, Jiang WT, Jean JS, Liu CC (2010). Cation exchange interaction between antibiotic ciprofloxacin and montmorillonite. J Hazard Mater.

[CR75] Wang CJ, Li Z, Jiang WT (2011). Adsorption of ciprofloxacin on 2:1 dioctahedral clay minerals. Appl Clay Sci.

[CR76] Wang L, Chen G, Ling C, Zhang J, Szerlag K (2017). Adsorption of ciprofloxacin on to bamboo charcoal: effects of pH, salinity, cations, and phosphate. Environ Prog Sustain Energy.

[CR77] Wang J, Liu G, Li T, Zhou C (2015). Physicochemical studies toward the removal of Zn(ii) and Pb(ii) ions through adsorption on montmorillonite-supported zero-valent iron nanoparticles. RSC Adv.

[CR78] Wang Y, Shen C, Zhang M, Zhang BT, Yu YG (2016). The electrochemical degradation of ciprofloxacin using a SnO2-Sb/Ti anode: influencing factors, reaction pathways and energy demand. Chem Eng J.

[CR79] Weber WJ, Morris JC (1963) Kinetics of adsorption on carbon from solution. J Sanit Eng Div 89(2):31–60

[CR80] Wu S, Zhao X, Li Y, Zhao C, du Q, Sun J, Wang Y, Peng X, Xia Y, Wang Z, Xia L (2013). Adsorption of ciprofloxacin onto biocomposite fibers of graphene oxide/calcium alginate. Chem Eng J.

[CR81] Wu M, Zhao S, Jing R, Shao Y, Liu X, Lv F, Hu X, Zhang Q, Meng Z, Liu A (2019). Competitive adsorption of antibiotic tetracycline and ciprofloxacin on montmorillonite. Appl Clay Sci.

[CR82] Xiao X, Zeng X, Lemley AT (2010). Species-dependent degradation of ciprofloxacin in a membrane anodic fenton system. J Agric Food Chem.

[CR83] Xu X, Liu Y, Wang T, Ji H, Chen L, Li S, Liu W (2019). Co-adsorption of ciprofloxacin and Cu(II) onto titanate nanotubes: speciation variation and metal-organic complexation. J Mol Liq.

[CR84] Yin D, Xu Z, Shi J, Shen L, He Z (2018). Adsorption characteristics of ciprofloxacin on the schorl: kinetics, thermodynamics, effect of metal ion and mechanisms. J Water Reuse Desalin.

[CR85] Zeng ZW, Tan XF, Liu YG, Tian SR, Zeng GM, Jiang LH, Liu SB, Li J, Liu N, Yin ZH (2018) Comprehensive adsorption studies of doxycycline and ciprofloxacin antibiotics by biochars prepared at different temperatures. Front Chem:6. 10.3389/fchem.2018.00080 10.3389/fchem.2018.00080 PMC5880934 29637067

[CR86] Zhou GJ, Lin L, Li XY, Leung KMY (2020). Removal of emerging contaminants from wastewater during chemically enhanced primary sedimentation and acidogenic sludge fermentation. Water Res.

