# Supplementary material for: Characterization of activated bentonite clay mineral and the mechanisms underlying its sorption for ciprofloxacin from aqueous solution
Source: Environ Sci Pollut Res Int. 2020 Jun 10;27(26):32980–97. doi: 10.1007/s11356-020-09267-1 (PMC7417422; doi:10.1007/s11356-020-09267-1)
Supplement: Supplementary file 1 — (DOCX 49 kb) [file 11356_2020_9267_MOESM1_ESM.docx]

**Characterization of activated bentonite clay mineral and the mechanisms underlying its sorption for ciprofloxacin from aqueous solution**

Ali Maged^a,b,*^, Sherif Kharbish^b^, Ismael Sayed Ismael^b^, Amit Bhatnagar^a,*^

*^a^ Department of Environmental and Biological Sciences, University of Eastern Finland, P.O. Box 1627, FI-70211 Kuopio, Finland*

*^b^* *Geology Department, Faculty of Science, Suez University, El Salam City, Suez Governorate 435 18, Egypt*

*Corresponding author: Ali Maged ([Ali.Maged@suezuni.edu.eg](mailto:Ali.Maged@suezuni.edu.eg))

*Corresponding author: Amit Bhatnagar (amit.bhatnagar@uef.fi)

*S1. the optimization of acid activation*

The effect of HCl concentration on the modification of bentonite was investigated (**Fig. S1**). HCl concentration was 0.25, 0.5, 0.75, 1.0, 1.25 and 1.50 mol/L.

**Fig. S1**. Effect of acid activation of bentonite on the removal of CIP molecules

*S2. Physicochemical analysis*

The mineralogical analysis of NB and AAB samples was carried out using a powder X-ray diffractometer (model XRD-6100, Shimadzu, Japan) in 2θ range of 3 to 70° with Cu-Kα radiation (λ = 1.5405 Å). The interlayer space (d-values) of the bentonite samples was calculated according to Bragg´s law (Bragg and Bragg 1913) using the following Eq. (S1).

$n \lambda=2d\sin\theta$ (S1)

The functional groups before and after the acid activation of bentonite were determined using Fourier transform infrared (FT-IR) analysis (model Cary 660 FTIR spectrometer, Agilent technologies, USA). The FTIR analysis of NB and AAB samples was performed within the range of 4000–600 cm-1 by accumulating 256 scans at a resolution of 4 cm-1. The changes in the surface morphology of NB and AAB were analyzed using Scanning Electron Microscopy (SEM) analysis (model TESCAN VEGA-LMU, Czech Republic) within magnification range of 500–5000 x. The alteration in chemical composition before and after the acid activation of the bentonite sample was investigated using Energy-dispersive X-ray spectroscopy (EDX) analysis (model INCAx-act, Oxford Instruments, UK). The elemental analysis of NB and AAB samples was performed using X-ray fluorescence (XRF) (model Philips PW 1410). The evaluation of the specific surface area of bentonite before and after the acid activation was performed using the N2 adsorption/desorption analysis (model Belsorp Mini II, Japan) at -196 °C. The point of zero charge (pHzpc) of NB and AAB was determined by following previously reported procedure by Maged et al., (2019). Briefly, NaCl solution (10 mM) was adjusted in the range of pH 2 – 10. A desired amount (0.05 g) of adsorbent was dispersed in 25 mL of the previously adjusted pH solutions and then agitated for 24 h at 200 rpm in an orbital shaker. Thereafter, the final pH was measured and the deviation in pHs was recorded. pHzpc was determined at the point which the final pH was equal to the initial pH. The CIP concentrations in different solutions were analyzed by a double beam UV–visible spectrophotometer (model UV-2401PC, Shimazdu Corporation, Japan) at wavelength 277 nm.

*S3. Theoretical Models*

*S3.1. Kinetic Modelling*

Kinetic studies of pollutant removal by an adsorbent is important for evaluating the rate and mechanism of adsorption. In this study, three models namely pseudo-first order, pseudo-second order and Avrami model were applied to the experimental data of kinetic studies. The equation for pseudo-first order kinetics was introduced initially by Lagergren (Lagergren 1898), and it is generally expressed as Eq. (S2). The formula for pseudo-second order kinetics is generally employed in the form proposed by Ho and McKay (Ho and McKay 1999) as expressed in Eq. (S3). intra-particle diffusion (IPD) (Weber and Morris 1963) in Eq. (S4). These models were fitted to the experimental data by non-linear modeling. The model’s parameters were measured and optimized by non-linear regression modeling by MATLAB (R2018b).

${q_{t}=q}_{e}(1-e^{-k_{1}t}$) (S2)

$q_{t}= \frac{k_{2}q_{e}^{2}t}{1+K_{2}q_{e}t}$ (S3)

${(q}_{t}=k_{\mathrm{id}}t^{1/2}+C\boldsymbol{)}$) (S4)

where, $q_{e}$ and $q_{t}$ (mg/g) are the adsorption capacity at equilibrium and at time t (min) respectively, $k_{1}$ (1/min) is the rate constant of pseudo-first order kinetics, $k_{2}$ (g/mg min) is the rate constant of the pseudo-second order kinetics and $k_{id}$ is IPD constants (mg/g min^1/2^).

*S3.2. Isotherm modeling*

In general, the adsorption isotherm represents adsorbent and adsorbate interaction at variable initial concentrations. In this study, three isotherm models were selected to describe the adsorption of CIP onto NB and ABB. These include two-parameters (Langmuir and Freundlich) and three-parameters (Sips) isotherm models.

The Langmuir (Langmuir 1918), Freundlich (Freundlich 1924) and Sips (Sips 1948) isotherm models were fitted with the experimental data by non-linear modeling using Eqs. (S5-7). The model’s parameters were measured and optimized by non-linear regression modeling by MATLAB (R2018b).

$q_{e}=\frac{q_{m}K_{L}C_{e}}{1+K_{L}C_{e}}$ (S5)

${q_{e}=K}_{F}C_{e}^{\frac{1}{n}}$ (S6)

$q_{e}=\frac{K_{s}C_{e}^{\beta s}}{1+a_{s}C_{e}^{\beta s}}$ (S7)

where, $q_{e}$ (mg/g) is the adsorption capacity of NB and AAB at the equilibrium time, $C_{e}$ is the equilibrium concentration (mg/L), $q_{m}$ (mg/g) is the Langmuir constant associated with adsorption capacity at its maximum adsorption capacity (mg/g), $k_{L}$ is the affinity between the sorbent and sorbate (L/mg), $k_{F}$ is the Freundlich constant (mg/g), $n$ is the Freundlich exponent related to adsorption intensity and $K_{s}$ (L/mg) is the Sips isotherm model constant.

The correlation coefficient (*R*^2^) was used to indicate the best fit of the experimental data with the models. Two different indicators were used for comparing different models that represent similar dependent variables, correlation coefficient (*R*^2^) and root mean square error (RMSE). The model which has a RMSE with a lower value defines the data more precisely than a model with larger values of this indicator The RMSE can be calculated using Eq. (S8) (Hafshejani et al. 2017).

$RMSE= \sqrt{\frac{\sum_{1}^{N} ( q_{exp}-q_{model} )^{2}}{N}}$ (S8)

where, $q_{exp}$ and $q_{model}$ are the experimentally measured value and model prediction for CIP sorption, respectively.

**References**

Bragg WH, Bragg WL (1913) The Reflection of X-rays by Crystals. Proc R Soc A Math Phys Eng Sci 88:428–438. doi: 10.1098/rspa.1913.0040

Freundlich H (1924) Kolloidchemie und Biologie. Naturwissenschaften 12:233–239. doi: 10.1007/BF01505512

Hafshejani LD, Tangsir S, Daneshvar E, et al (2017) Optimization of fluoride removal from aqueous solution by Al2O3 nanoparticles. J Mol Liq 238:254–262. doi: 10.1016/J.MOLLIQ.2017.04.104

Ho Y., McKay G (1999) Pseudo-second order model for sorption processes. Process Biochem 34:451–465. doi: 10.1016/S0032-9592(98)00112-5

Lagergren (1898) About the Theory of So-called Adsorption of Soluble Substances. Sven Vetenskapsakad Handingarl 24:1–39

Langmuir I (1918) The adsorption of gases on plane surfaces of glass, mica and platinum. J Am Chem Soc 40:1361–1403. doi: 10.1021/ja02242a004

Maged A, Iqbal J, Kharbish S, et al (2019) Tuning tetracycline removal from aqueous solution onto activated 2:1 layered clay mineral: characterization, sorption and mechanistic studies. J Hazard Mater 121320. doi: 10.1016/J.JHAZMAT.2019.121320

Sips R (1948) On the Structure of a Catalyst Surface. J Chem Phys 16:490–495. doi: 10.1063/1.1746922

Weber WJ, Morris JC (1963) Kinetics of adsorption on carbon from solution. Kinet Adsorpt Carbon from Solut
